# Salivary proteomics of healthy dogs: An in depth catalog

**DOI:** 10.1371/journal.pone.0191307

**Published:** 2018-01-12

**Authors:** Sheila M. F. Torres, Eva Furrow, Clarissa P. Souza, Jennifer L. Granick, Ebbing P. de Jong, Timothy J. Griffin, Xiong Wang

**Affiliations:** 1 Veterinary Clinical Sciences Department, College of Veterinary Medicine, University of Minnesota, Saint Paul, Minnesota, United States of America; 2 Clinical Sciences Department, College of Veterinary Medicine and Biomedical Sciences, Colorado State University, Fort Collins, Colorado, United States of America; 3 Department of Biochemistry, Molecular Biology and Biophysics, University of Minnesota, Minneapolis, Minnesota, United States of America; 4 Department of Biochemistry and Molecular Biochemistry, SUNY Upstate Medical University, Syracuse, New York, United States of America; 5 Department of Veterinary Biomedical Sciences, University of Minnesota, Saint Paul, Minnesota, United States of America; 6 Minnesota Department of Health, Saint Paul, Minnesota, United States of America; Nanjing Medical University, CHINA

## Abstract

**Objective:**

To provide an in-depth catalog of the salivary proteome and endogenous peptidome of healthy dogs, evaluate proteins and peptides with antimicrobial properties, and compare the most common salivary proteins and peptides between different breed phylogeny groups.

**Methods:**

36 healthy dogs without evidence of periodontal disease representing four breed phylogeny groups, based upon single nucleotide polymorphism haplotypes (ancient, herding/sighthound, and two miscellaneous groups). Saliva collected from dogs was pooled by phylogeny group and analyzed using nanoscale liquid chromatography-tandem mass spectrometry. Resulting tandem mass spectra were compared to databases for identification of endogenous peptides and inferred proteins.

**Results:**

2,491 proteins and endogenous peptides were found in the saliva of healthy dogs with no periodontal disease. All dog phylogeny groups’ saliva was rich in proteins and peptides with antimicrobial functions. The ancient breeds group was distinct in that it contained unique proteins and was missing many proteins and peptides present in the other groups.

**Conclusions and clinical relevance:**

Using a sophisticated nanoscale liquid chromatography-tandem mass spectrometry, we were able to identify 10-fold more salivary proteins than previously reported in dogs. Seven of the top 10 most abundant proteins or peptides serve immune functions and many more with various antimicrobial mechanisms were found. This is the most comprehensive analysis of healthy canine saliva to date, and will provide the groundwork for future studies analyzing salivary proteins and endogenous peptides in disease states.

## Introduction

Saliva is composed of a complex mixture of enzymes, glycoproteins, immunoglobulins, peptides, inorganic substances, white blood cells, epithelial cells, and microflora, in addition to water. The substances in saliva originate primarily from salivary glands but also blood and nasal-bronchial secretions [1–2]. Food digestion and lubrication are well-recognized functions of saliva; however, this complex fluid also protects the oral cavity against pathogens, maintains the mouth pH and has a role in taste [3–4].

For many decades saliva has been considered the reflection of health and disease states of the oral cavity in addition to the whole body [4]. The relatively easy and non-invasive access to saliva samples compounded with the remarkable advances in the technology to investigate proteins—a major saliva component—have spiked researchers’ interest in looking at the composition of this biological fluid in healthy individuals with the ultimate goal of identifying biomarkers of diseases [4–9].

Various mass spectrometry methods are currently available and have been widely used to study the salivary proteins, and smaller, endogenous peptides. However, despite extensive investigation in humans [2,5,8,10–26], much less work has been done in other species [3,27–34]. Recently, the protein components of dog saliva were examined using SDS-PAGE-LC coupled to tandem mass spectrometry (MS/MS) [3], but only one dog sample was analyzed. Hence, a more comprehensive characterization of the salivary proteome and endogenous peptidome of healthy dogs is needed and can provide a valuable groundwork for future studies searching for specific changes in salivary protein composition associated with oral and systemic diseases.

The primary study aim was to provide an in-depth catalog of the salivary endogenous peptidome and proteome of healthy dogs. Additional aims included an evaluation of proteins and peptides with antimicrobial properties and comparison of the most common salivary proteins between different breed phylogeny groups.

## Materials and methods

**Fig 1:**
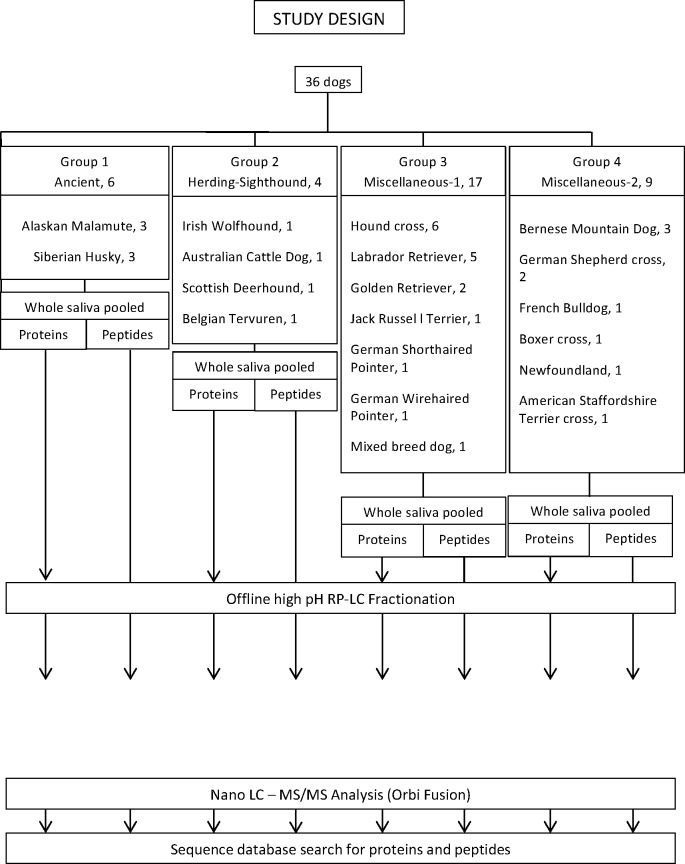
Flow chart summarizing the study design.

A summary of the study design and methodology used is shown in Fig 1.

### Animals

The study was approved by the Institutional Animal Care and Use Committee of the University of Minnesota. Thirty-six clinically healthy dogs owned by faculty, staff and students at the University of Minnesota, College of Veterinary Medicine, were selected for the study. The dogs were carefully examined by a veterinarian with experience in dental disease to assure the absence of any dental abnormalities, especially periodontal disease. Sixteen dogs were males (11 intact and 5 neutered) and 20 were females (16 spayed and 4 intact). Their age ranged from 4 to 148 months (mean = 40.58 months; median = 25 months). The following breeds were represented: Hound cross (n = 6), Labrador Retriever (5), Alaskan Malamute (3), Bernese Mountain Dog (3), Siberian Husky (3), Golden Retriever (2), German Shepherd cross (2), American Staffordshire Terrier cross (1), Australian Cattle Dog (1), Belgian Tervuren (1), Boxer cross (1), French Bulldog (1), German Shorthair Pointer (1), German Wirehair Pointer (1), Irish Wolfhound (1), Jack Russell Terrier (1), Mixed breed dog (1), Newfoundland (1), and Scottish Deerhound (1) (Fig 1).

To try to investigate the potential effect of genetic lineage on the salivary proteomics and peptidomics, the dogs were initially selected to represent the four lineages that cluster based on structure analysis of microsatellite markers: asian/ancient, herding, hunting, and mastiff [35]. However, more recent analysis of single nucleotide polymorphism haplotypes divides dog breeds into 10 clusters [36]. Given this newer classification and the inclusion of some dogs with incomplete parentage information (crossbred dogs), the four groups were renamed as follows: ancient, herding-sighthound, and miscellaneous 1 and 2 (Fig 1). Saliva was pooled for dogs within each group.

### Saliva collection

Whole saliva was collected without previous stimulation using saliva collection kits (SalivaBio kit; Salimetrics^®^, State College, PA) according to the manufacturer’s recommendation. Briefly, dog owners were asked to withhold food and water from their pets for at least 1 hour before sample collection. A cotton swab of 125mm length and 8mm diameter was placed in each dog’s cheek pouches for 45–60 seconds, with the collector gently holding the dog’s muzzle to prevent swallowing. Upon removal, the swab was placed in a special tube (Swab Storage Tube) and the saliva extracted by centrifugation at 685 × *g* for 15 minutes. The swab was removed from the tube and the saliva immediately stored at -80°C freezer until analysis [37].

### Sample preparation (offline high pH reverse phase-liquid chromatography fractionation)

Saliva samples were thawed on ice and cleared of cells and debris by centrifugation at 3000 × *g* for 10 minutes and 16,100 × *g* for 1 minute at 4°C. Protein concentrations of the supernatants were determined by the bicinchoninic acid (BCA) assay. All samples were qualitatively analyzed by SDS-PAGE prior to being used in the study.

Pools of saliva from each group were prepared for proteomic analysis by combining equal protein amounts for each sample within a category to a total protein amount of 200 μg per pool. The pools were digested using the FASP protocol [38] using 10kDa filters (Pall Nanosep 10kDa filters; VWR, OD010C34) and the resulting peptides desalted using silica-based sorbent cartridges (Sep-Pak tC18 cartridges; Waters, WAT054925).

Endogenous peptides (naturally occurring salivary peptides below 10kDa) were collected from the flow-through after the initial centrifugation step using the 10kDa filters (prior to protein alkylation). These peptides were separately reduced using 5 mM tris (2-carboxyethyl) phosphine (TCEP) and alkylated using 50 mM iodoacetamide. Endogenous peptide samples were cleaned up on mixed-mode polymeric sorbent cartridges (Oasis MCX cartridges; Waters, 186000253).

The trypsin-digested protein samples were fractionated using high-pH reversed phase with subsequent concatenation similar to the protocol of Wang et al [39]. The samples were dissolved in 200 mM ammonium formate, pH 10 containing 2% acetonitrile (ACN) and loaded onto a C18 column (Phenomenex Kinetek C18 column; 2.6 μm, 2.1 x 100 mm). Solvents A and B were 20 mM ammonium formate, pH 10, containing 2 and 90% ACN respectively. A gradient was run at 200 μL/min with the following steps: 0 min, 2% B; 5 min, 2% B; 5.5 min, 5%B; 28 min, 30% B; 31 min, 60% B; 33 min, 90% B; 40 min, 90% B; 41 min, 2% B; 45 min, 2% B. The column was heated to 55°C with a heated sleeve (Analytical Sales & Service, Inc., HSI-25L). Fractions were collected every minute, and were concatenated by combining a volume equivalent to 15 mAU from fractions 7 and 21, 8 and 22, etc., until fractions 20 and 34 to produce 14 concatenated fractions. These were dried in a speed-vac, and re-dissolved in 37.5 μL of 0.1% trifluoroacetic acid (TFA) in 2% ACN load solvent.

### Nano liquid chromatography–tandem mass spectrometry (LC-MS/MS) analysis

Analysis of the concatenated fractions from the digest proteins, as well as the endogenous peptides, was performed on a mass spectrometer (Orbitrap Fusion Tribrid with Easy-nLC autosampler and LC; Thermo Scientific, Waltham, MA) equipped with an autosampler and LC system. For protein samples, 2.5 μL of each concatenated fraction was injected directly onto an in-house packed, 10 cm x 75 μm column packed with 3 μm C18 particles. Separation was achieved by a gradient from 2–30% B over 50 min, followed by a 2 min ramp to 90% B and 8 min hold at 90% B. The flow rate was 200 nL/min. The MS operated in a top speed, data-dependent mode with a cycle time of 3 s. MS1 scans were performed in the Orbitrap at 120k resolution from 400–1500 m/z with an AGC target of 4E5. Percursor isolation took place in the quadrupole with an isolation width of 1.6 m/z. CID was performed at 35% NCE and MS2 spectra were collected in the ion trap. Dynamic exclusion used a repeat count of 1 for a duration of 30 s.

### Sequence database search for proteins and peptides

The data were searched against a RefSeq Canis familiaris database with common contaminant proteins, containing 47336 entries, using protein analysis software (Sequest HT node in Proteome Discoverer 2.0; Thermo Scientific, Waltham, MA). Search parameters used included trypsin enzyme specificity with ≤2 missed cleavages for analysis of intact proteins, carbamidomethyl as a fixed modification on cysteine and variable modification of methionine oxidation and protein N-terminal acetylation. Precursor and product ion mass tolerances of 35 ppm and 0.6 Da were used. For identification of endogenous peptides, all parameters were the same as above, except that no enzyme was specified.

### Criteria for protein identification

MS/MS based peptide and inferred protein identifications were validated using proteomic analysis software (Scaffold; version Scaffold 4.6.1; Proteome Software Inc., Portland, OR). For the analysis of intact salivary proteins, peptide identifications were accepted if they could be established at greater than 92.0% probability by the Scaffold Local FDR algorithm. Peptide identifications were also required to exceed specific database search engine thresholds. Sequest identifications required at least deltaCn scores of greater than 0.0 and XCorr scores of greater than 1.8, 2.2, 2.5 and 3.5 for singly, doubly, triply and quadruply charged peptides. Protein identifications were accepted if they could be established at greater than 5.0% probability to achieve an estimated FDR less than or equal to 1.0% and contained at least 1 identified peptide. Protein probabilities were assigned by the Protein Prophet algorithm [40]. Proteins that contained similar peptides and could not be differentiated based on MS/MS analysis alone were grouped to satisfy the principles of parsimony. Proteins sharing significant peptide evidence were grouped into clusters. Collectively, the above criteria resulted in an estimated peptide FDR of 0.2% and an estimated protein FDR of 1.0%, both estimated using the target-decoy method. S1 Appendix contains all information on identified proteins and peptides for the analysis of intact salivary proteins.

For the identification of endogenous salivary peptides, accepted peptide identifications were stringently filtered to an estimated FDR level of 0.0% using the target-decoy method for estimation. S2 Appendix contains all information on identified endogenous peptides.

### Bioinformatics analyses

The semi-quantitative protein data from the dog saliva was measured via protein spectral counts from the MS-based proteomics data. Normalized spectral counts were assigned to each identified protein using the “Quantitative Value” assignment tool within the Scaffold software used for organizing protein identifications and comparing spectral counts across samples. Quantitative values for identified proteins and endogenous peptides were compared across the four groups.

To be included in the analyses each specific protein and peptide had to meet at least one of the following criteria: (i) an assigned normalized spectral count value of 5 or higher in at least one group or (ii) a normalized spectral count value of 1 or more in at least two groups. After application of the criteria in the dataset, the gene symbols of included proteins were imported into a web-based program (Venny 2.1) for construction of Venn’s diagram [41] with the goal of comparing the protein and peptide content among groups. A two dimensional Principle Component Analysis (PCA) and Heatmap were also generated using a web tool for visualizing multivariate data (ClustVis) [42] to further evaluate similarities and differences in the salivary proteomics and peptidomics among the four groups.

### Search of proteins and peptides with immune functions

Papers on salivary proteomics/peptidomics were reviewed to identify proteins and peptides with antimicrobial functions that are reported to be abundant in human saliva (specific proteins and references are provided in the results); our database was then searched for these proteins and peptides.

## Results

### Dog saliva proteomic and peptidomic profile

Using nanoscale LC MS/MS 2,491 proteins and endogenous peptides were identified in the dog saliva (S3 Appendix), and 1,588 of those met the defined quantitative criteria for further analysis. The top 10 most abundant proteins and their function are provided in **Table 1**; 7 of the 10 have immune functions. **Table 2** additionally shows salivary proteins and peptides with various antimicrobial properties that have been reported to be abundant in previous studies. Most of these proteins and peptides were present in all four groups.

**Table 1:** Most abundant canine salivary proteins and their functions.

Protein name	Gene name	Total normalized spectral counts	Function [reference]
Fc fragment of IgG binding protein	FCGBP	797	Binds to IgG on mucosal surfaces [43]
Polymeric immunoglobulin receptor (precursor)	PIGR	571	Transports IgA across epithelial cells [44]
BPI fold-containing family A member 2	BPIFA2	466	Key components of the innate immune response against Gram-negative bacteria [45]
BPI fold-containing family B member 1 isoform X2	BPIFB1	352	Key components of the innate immune response against Gram-negative bacteria [45]
Albumin	ALB	296	Serum-derived protein believed to passively enter saliva. Saliva-specific functions include binding to hydroxyapatite and lubrication of oral tissues [46]
Ovostatin homolog 2-like	LOC611455	270	GO: serine-type endopeptidase inhibitor activity
Mucin 19	MUC19	264	Gel-forming mucin that lubricates saliva and plays a role in reducing adherence and increasing clearance of bacteria [47]
Angiopoietin-related protein 5-like	LOC607055	261	Not reported
Actin gamma 1	ACTG1	244	Cytoskeletal protein with multiple functions in the defense against intracellular pathogens [48]
Ig lambda chain V-I region BL2	LOC607368	219	Component of immunoglobulin light chains

**Table 2:** Canine salivary proteins and peptides with antimicrobial properties.

Protein name	Gene name	Normalized spectral counts per dog group*	Function [reference]
Antileukoproteinase or secretory leukocyte protease inhibitor (precursor)	SLPI	4-2-2-1	The N-terminal cationic domain has an antibacterial, antifungal and antiviral effect [49–51]
Beta 2 microglobulin (precursor)	B2M	0-1-2-2	Agglutinates bacteria (e.g. *Streptococcus mutans*) [50–52]
BPI fold-containing family A member 2	BPIFA2	41-136-103-186	Key components of the innate immune response against Gram-negative bacteria [45]
BPI fold-containing family B member 1 isoform X2	BPIFB1	42-96-67-147	Key components of the innate immune response against Gram-negative bacteria [45]
Carbonic anhydrase 6 isoform X1	CA6	16-42-50-69	Binds to *Staphylococcus aureus* [53]
Cathelicidin antimicrobial peptide (precursor)	CAMP	1-1-1-2	Antibacterial and antifungal effects by disruption of cell membrane. It also binds and neutralizes lipopolysaccharide from Gram-negative bacteria [50,54,55]
Cystatin-M	CST6	2-7-9-8	Cystatins block the action of bacterial proteases [49,50,56]
Cystatin-A	CSTA	2-2-0-1	
Deleted in malignant brain tumors 1 protein isoform X1	DMBT1	7-27-27-44	Known as salivary agglutinin and is identical to Gp-340 expressed in lungs. Binds to a wide variety of microorganisms [49,50,53,57]
Elafin/skin-derived antileukoproteinase (SKALP) (precursor)	PI3	1-1-0-0	Kills Gram-negative and Gram-positive bacteria [50,58]
Fibronectin (partial, predicted)	FN1	6-12-20-8	Agglutinates bacteria and prevents its adhesion to oral surfaces [50,57]
Immunoglobulin J chain isoform 1 (predicted)	IGJ	0-3-10-4	Binds to *Staphylococcus aureus* [53]
Lactotransferrin (Precursor)	LTF	8-43-52-80	Bacteriostatic due to its iron-depriving effects [49,50]
Lactoperoxidase isoform 2	LPO	3-41-37-63	Catalysis the formation of bactericidic compounds [49,50]
Lysozyme C, milk isozyme-like (predicted)	LYZF2	3-55-72-63	Defense response to bacterium; regulation of macrophage activation; lysis bacteria cell wall polysaccharides; activates bacterial autolysins [49,50,53]
Lysozyme precursor (cluster)	LYZ	28-24-19-28	
Mucin-5B (predicted)	MUC5B	14-46-69-72	Modulates the microbial colonization of oral epithelial surfaces [49]
Mucin-7 (predicted)	MUC7	0-0-1-2	Binds to a variety of bacteria [49,50,59–61]
Mucin-19 (predicted)	MUC19	0-65-93-106	Gel-forming mucin that lubricates saliva and plays a role in reducing adherence and increasing clearance of bacteria [47]
Myeloperoxidase	MPO	6-3-7-4	Catalyses the hydrogen peroxide oxidation of thiocynate ions which forms the bactericidal product, hypothiocyanite [50]
Peptidoglycan recognition protein 1	PGLYRP1	4-3-2-2	It binds to the bacterial cell wall peptidoglycans to exert the bactericidal effect, but do not permeabilize bacterial membranes. They are bactericidal for Gram-positive bacteria and bacteriostatic for Gram-negative bacteria [50,62,63]
Polymeric immunoglobulin receptor (precursor)	PIGR	2-164-204-201	Transports IgA across epithelial cells [44]
Serpin B10 (predicted)	SERPINB10	3-8-9-14	Positive regulation of defense response to virus by host
S100-A8	S100A8	2-5-5-11	Also known as calgranulin A (S100-A8) and B (S100-A9). The dimer of calgranulin A and B is called calprotectin is expressed in neutrophils, macrophages and keratinocytes cytosols. They inhibit bacterial growth by scavenging divalent cation [50,64]
S100-A9 isoformX3	S100A9	2-4-1-8	
Zymogen granule protein 16 homolog B	ZG16B	1-5-6-15	Binds to *Staphylococcus aureus* [53]

* Groups are sequentially as follows: Ancient, Herding-Sighthound, Miscellaneous-1,Miscellaneous-2

### The salivary proteomic and peptidomic content of the Ancient group stands out from other breed groups

**Fig 2:**
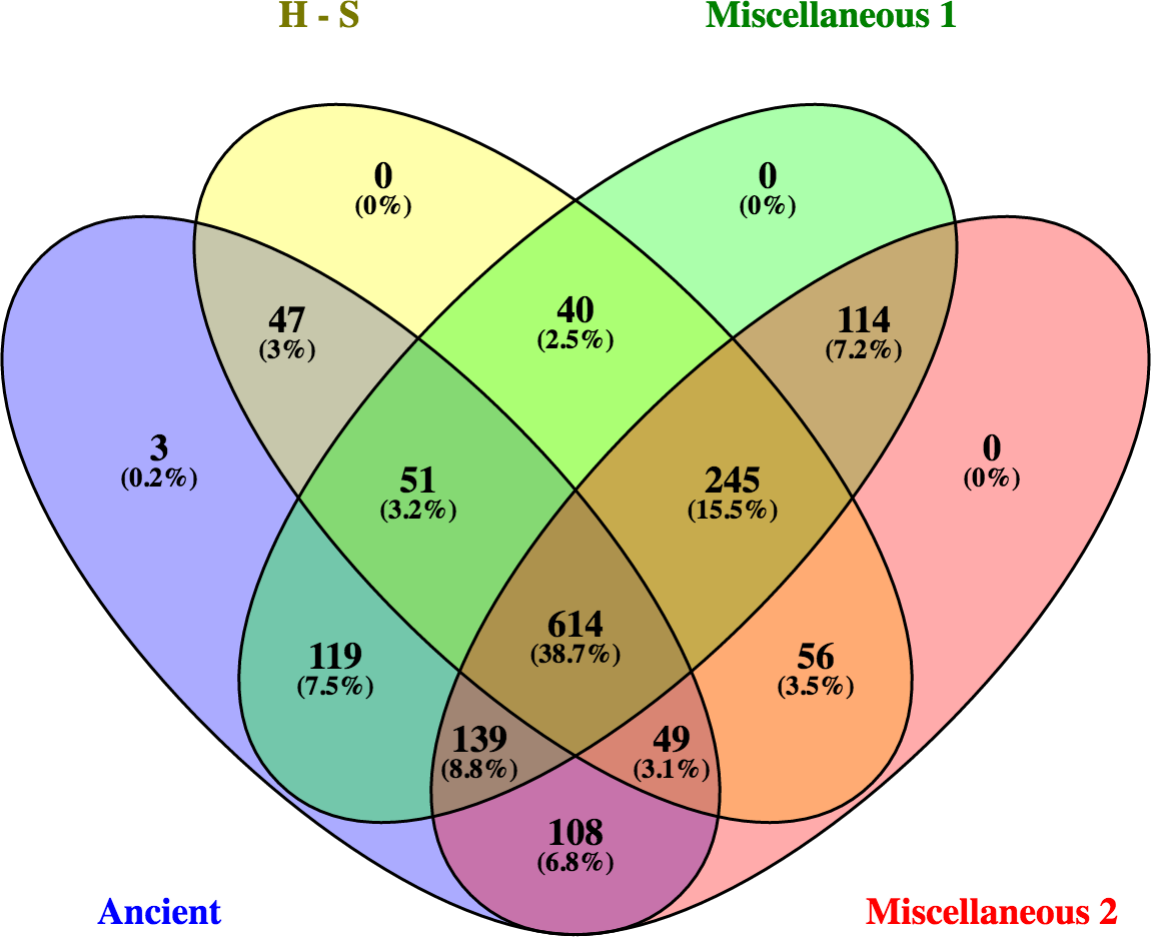
Venn diagram displaying the overlapped and unique proteins among the four groups. H-S = Herding-Sighthound.

Comparative analyses using a Venn diagram showed that the four groups shared 614 proteins and peptides representing 38.7% of the analyzed proteins/peptides (Fig 2). In contrast to the other three groups, unique proteins (SPTBN2 (normalized spectral count = 7), TMOD3 (6) and PSMC4 (5)) were identified only in the Ancient group. In addition, there were 245 (15.5%) proteins that were shared by the Herding-Sighthound and Miscellaneous 1 and 2 groups, but not the Ancient group (Fig 2).

**Fig 3:**
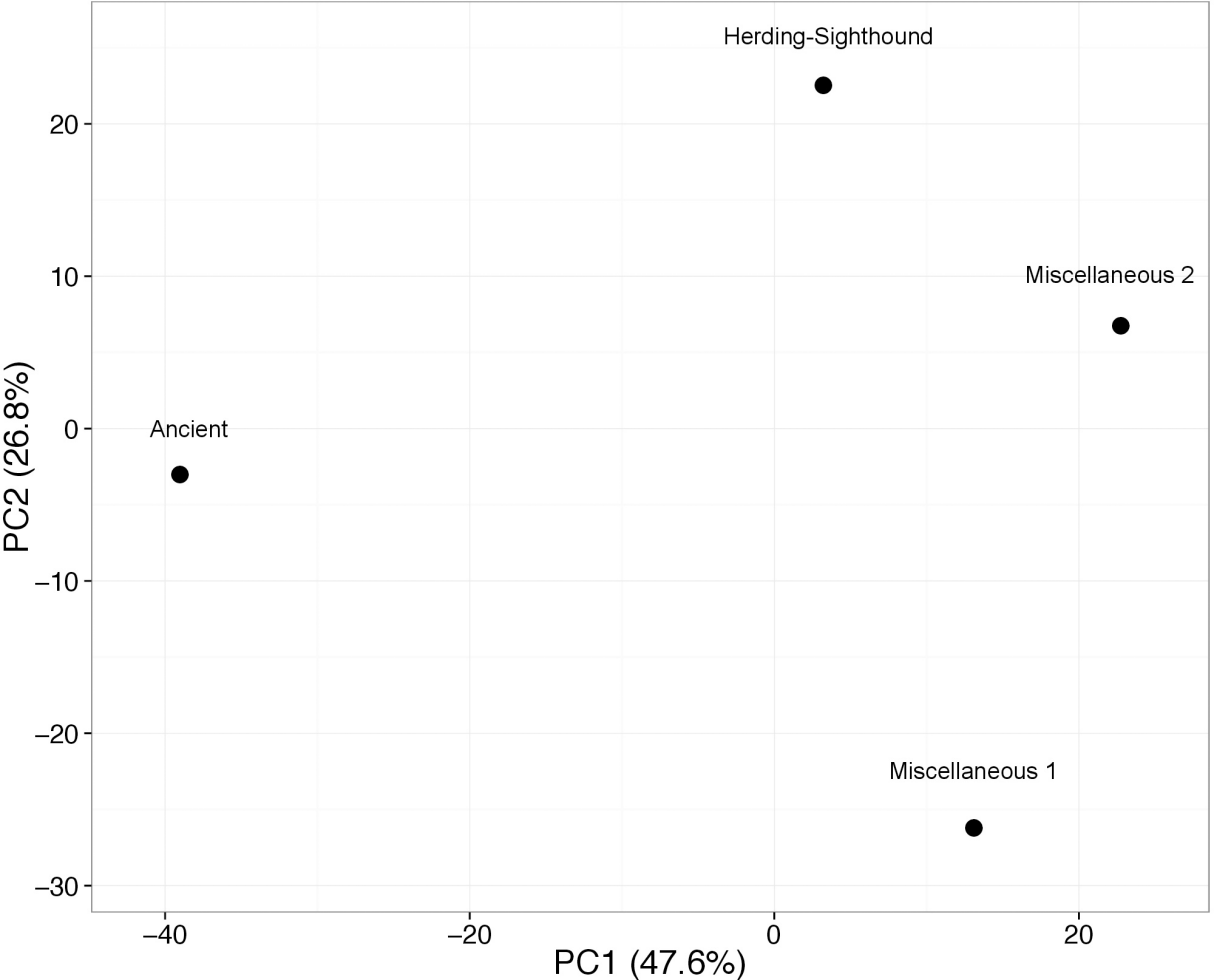
Two dimensional principal coordinate analysis showing the salivary protein and peptide profile relationship among the groups.

The unique protein profile in the Ancient group was also reflected in the PCA analysis (Fig 3). The Ancient group nested distant from the other three groups at the far end of PC1 (X-axis), which explained 47.6% of the total variance. When taking into consideration PC2 (Y-axis), which explained 26.8% of total variance, Herding-Sighthound and Miscellaneous group 2 clustered more closely than Miscellaneous group 1 and Ancient group.

**Fig 4:**
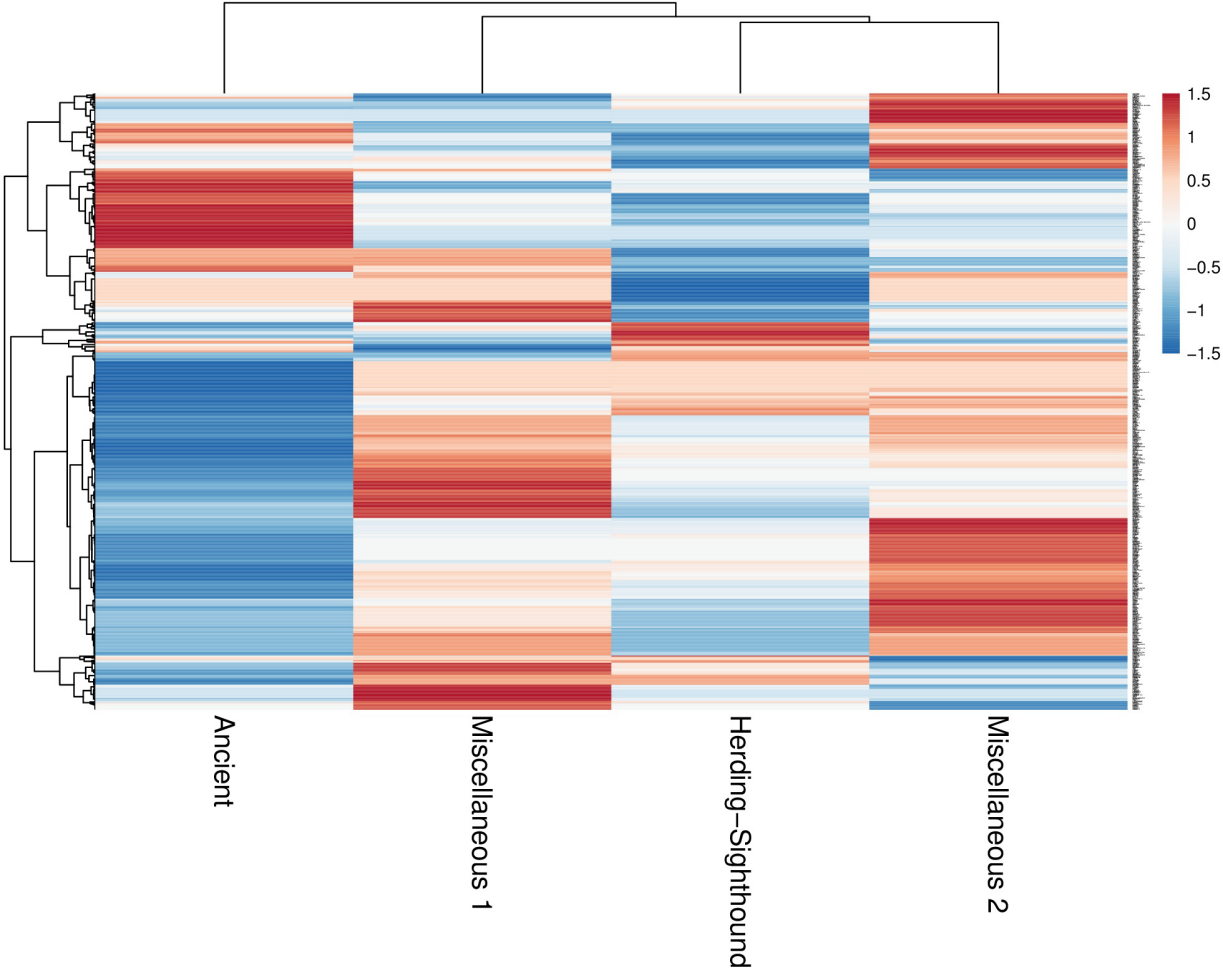
Heatmap showing the relative abundance (color) and relationship (dendogram) of salivary proteins and peptides among the groups.

The intergroup relationship in the PCA analysis is also confirmed by the dendrogram in Fig 4, which shows the relationship of the four groups. The predominance of blue color in the Ancient group indicates relatively less abundance of specific proteins and peptides in this group compared to the other groups.

## Discussion

Using nanoscale LC–MS/MS we identified 2,491 proteins and peptides in the saliva of healthy dogs with no periodontal diseases. In contrast, a recent study described only 244 proteins in dog saliva [3]. The substantially higher number of proteins identified in this study could be partially explained by differences in sample collection but a main contributor is most likely the mass spectrometer instrumentation used. For our study, we used a sophisticated mass spectrometer (Orbitrap Fusion Tribrid with Easy-nLC autosampler and LC (Thermo Scientific, Waltham, MA) for LC-MS/MS analysis which provides some of the highest sensitivity currently available for analysis of complex protein mixtures [65]. The prior study by de Sousa-Pereira and colleagues utilized an older generation MALDI-TOF/TOF instrument [3], which most likely explains the order of magnitude difference in proteins identified. It also highlights the depth of our study in terms of proteins and endogenous peptides identified, which provides a much more comprehensive view of the salivary proteome and peptidome in healthy dogs.

One of the most important functions of saliva is to protect the oral cavity and indirectly other organs against infections. In this study, 7 of the top 10 most abundant proteins have immune functions. Additionally, we identified 26 peptides and proteins (as well as some isoforms) that have been reported to have antimicrobial functions in human saliva; 4 of these were also in the top 10 most abundant in canine saliva. Six of the 26 proteins and peptides were not present in all four breed groups indicating the variability among individual dogs or dog breeds. There are likely many additional proteins and peptides with antimicrobial functions in the 2,491 identified in the study.

Antimicrobial peptides (AMP) are small molecular weight, typically cationic peptides that have a broad spectrum of action against bacteria, fungi, parasites and some viruses [66]. They are an important part of the innate immune response of almost all living organisms including plants, invertebrates and vertebrates and generally function by forming holes in the microorganisms’ cell membrane. Various AMP such as, alfa and beta defensins, cathelicidin, adrenomedullin, histatins, elafin, secretory leukocyte protease inhibitor (SLPI) and lysozyme have been found in human saliva [49,50,67]. Of these, we identified precursors of elafin, SLPI, cathelicidin and lysozyme and, de Sousa-Pereira and collaborators listed lysozyme in the saliva of the dog included in their study [3]. β-defensins are expressed in many epithelial tissues and have been identified in the skin of healthy dogs [68–70]. The absence of this category of AMP in the dog saliva was, to some extent, unexpected but, defensins were not reported in the saliva of dog, cattle, sheep, horse, rabbit and rat in a recent study [3]. Moreover, the lack of identification could be explained by their presence below the limit of detection of the method used. Differences in the expression of proteins and peptides in the saliva of humans and dogs could be partially explained by phylogenetic and dietary variations between these species. However, additional studies including a large number of dogs will be needed to corroborate our findings.

The dogs in this study were selected to represent diverse ancestral lineages both to provide a comprehensive dataset of the canine salivary proteome and peptidome and to determine if there were clear differences between breed groups. While only two of the four groups ultimately represented distinct genetic clusters based on the most recent canine genomics data, differences were evident, with only 38.7% of the analyzed proteins and peptides shared by all groups. The Ancient group, which included Siberian Huskies and Alaskan Malamutes, was the most distinct, and the only one with unique proteins. This parallels genetic differences in the breed groups; the breeds within the ancient group have a high level of divergence from other breeds. It cannot be determined from this data whether the unique proteins identified in the Ancient group are specifically characteristic of the northern group (the clade comprising the Siberian Husky and Alaskan Malamute) or if they are also a feature of saliva from other ancient breeds [36]. Future studies including a larger breed representation in the various phylogenic groups and a larger number of dogs per breed will help answer this question. Interestingly, a recent study showed unique proteins in the saliva of Korean people when compared to a comprehensive database of human salivary proteins indicating ethnic differences in the human saliva proteome [71].

In addition to genetic differences, other factors not investigated in this study could have also played a role in variations noted between the dog groups. Age, diurnal variation, health status and individual variation have all been shown to influence the composition of proteins in the saliva in humans [72–78]. These variables, and possibly others, most likely also impact the salivary protein and peptide profiles of dogs and need to be carefully and urgently investigated before we can obtain accurate information on changes in saliva protein and peptide components in disease states.

This study provides a comprehensive catalog of the proteins and endogenous peptides present in canine saliva. We included 36 dogs and divided them in groups based on breed phylogeny which revealed differences that parallel genetic clusters. Samples were pooled within each group, and this could have masked any inter-individual or gender variances in the salivary protein composition of the dogs. An important next step is to evaluate any possible influence of age, gender, breed and individual in the composition of proteins and peptides of the dog saliva.

## Supporting information

S1 Appendix Detailed information on identified proteins and peptides for the analysis of intact canine salivary proteins. (XLSX)

S2 Appendix Detailed information on identified endogenous peptides in canine saliva. (XLSX)

S3 Appendix Comprehensive list of protein and peptide genes identified in the saliva of dogs in each of the four groups. (XLSX)
